# Efficacy of Hypoxia Against *Tribolium castaneum* (Coleoptera: Tenebrionidae) Throughout Ontogeny

**DOI:** 10.1093/jee/toz019

**Published:** 2019-02-14

**Authors:** K Kharel, L J Mason, L L Murdock, D Baributsa

**Affiliations:** Department of Entomology, Purdue University, West Lafayette, IN

**Keywords:** hermetic technology, grain storage, hypoxia, controlled atmosphere, pest management

## Abstract

Hermetic grain storage technology offers a viable chemical-free approach to control storage insects. However, there is limited knowledge on how hypoxia affects the survival of insect life stages during grain storage in hermetic bags. We exposed *Tribolium castaneum* (Herbst) (Coleoptera: Tenebrionidae) eggs (2 d), young larvae (7 d), old larvae (21 d), pupae (28 d), and adults (2 d after emergence) to 2, 4, 8, and 20.9% oxygen levels for 1, 3, 5, 10, and 15 d and assessed subsequent mortality. At 2% oxygen, complete mortality was achieved in 3 d for eggs and young larvae, 10 d for old larvae and pupae, and 15 d for adults. At 4% oxygen, 15 d were required to kill all eggs and old larvae but not the other insect life stages. At 8% oxygen after 15 d, complete mortality of any insect life stage was not observed; but even a relatively short exposure (1–3 d) caused significant developmental delays in immature insects. Our study shows potential utility of hermetic technology for control of *T. castaneum*, but internal oxygen should be maintained below 2% level for at least 15 d for complete control. Increased oxygen levels improved the development of all insect life stages leading to increased adult emergence. There is a need to explore exposure time required to achieve complete mortality of all insect life stage above the 2% oxygen level.

*Tribolium castaneum* (Herbst) (Coleoptera, Tenebrionidae), the red flour beetle, is an important pest of stored cereal grains, pulses, and oilseeds. It has one of the highest population growth potentials for any stored-product insects (White 1988). *Tribolium castaneum* causes damage by feeding on the germ and endosperm of grain kernels and by contaminating the grain with body parts and feces. When the infestation level is high, *T. castaneum* secretes benzoquinones, which imparts a pungent odor to the commodity, rendering it unfit for consumption (Campbell and Runnion 2003). Pest control tactics for *T. castaneum* in stored grain rely heavily on synthetic insecticides, including slow-release grain fumigants such as phostoxin and essential oils. The grain may be treated with insecticidal dusts, residual sprays, or with other materials before storage (i.e., diatomaceous earth, Malathion, Chlorpyrifos-methyl) (Zettler and Cuperus 1990, Lee et al. 2002, Epidi and Odili 2009). Extensive use of chemicals has led *T. castaneum* to be resistant to many conventional insecticides (Jagadeesan et al. 2012). Furthermore, usage of insecticides on stored food commodities is a concern due to human and environmental health hazards associated with exposure, including danger to applicators using improper application practices.

Hermetic storage technology, a chemical-free approach, is a promising alternative for management of insect pests of stored grains and pulses. The technology reduces the oxygen supply of these pests by fostering the development of a hypoxic environment inside the storage system. Suboptimal levels of oxygen suppress insect population growth and survival. Hypoxic conditions are achieved mainly due to 1) respiration of insects and other organisms present including the grain itself and 2) making the storage system airtight to greatly restrict the movement of air from outside (Navarro et al. 1994). Studies have shown that hermetic bagging systems such as Purdue Improved Crop Storage (PICS) bags can maintain oxygen levels at 2–15% for extended periods of time, typically for 3–6 mo, which limits insect population growth (Murdock et al. 2012, Baoua et al. 2014, Kharel et al. 2018). Oxygen levels below 5% are effective in controlling various storage insects including *Callosobruchus maculatus* (Fabricius) (Coleoptera: Chrysomelidae), *Cryptolestes ferrugineus* (Stephens) (Coleoptera: Laemophloeidae), *Rhyzopertha dominica* (Fabricius) (Coleoptera: Bostrichidae), *Oryzaephilus surinamensis* (Linnaeus) (Coleoptera: Silvanidae), *Sitophilus oryzae* (Linnaeus) (Coleoptera: Curculionidae), *S. zeamais* Motschulsky (Coleoptera: Curculionidae), and *Trogoderma granarium* Everts (Coleoptera: Dermestidae) (Bailey 1965, Baributsa et al. 2010, Murdock et al. 2012, Baoua et al. 2014, Martin et al. 2015, Yan et al. 2016, Kharel et al. 2018). In the past, hermetic storage technology was mainly used by small-scale farmers to store their surplus crops for subsequent consumption or marketing. Thanks to its proven effectiveness against a broad range of storage pests, hermetic technology has begun to attract the attention of a wider circle of users such as large-scale farmers, grain traders, and development agencies.

Several studies have looked at the effects of low-oxygen on the mortality of *T. castaneum* eggs and adults (Navarro 1978, Tunc and Navarro 1983). Others have looked at the sensitivity of *T. castenaum* to modified atmospheres (low-oxygen, high carbon dioxide, and nitrogen) alone or in combination with temperatures (Donahaye et al.1992, 1996). These studies considered low-oxygen levels of 5% or below. No study has assessed the impact of a wide range of hypoxia levels that are realistically observable in hermetic bags during grain storage, on all life stages of *T. castaneum*. We hypothesized that the different developmental stages of insects might exhibit varying degrees of susceptibility to hypoxia observed in hermetic bags during grain storage. Better understanding of these effects could lead to improving the efficacy of hermetic storage for the control of *T. castaneum* and, by implication, other insects. Here we report the results of a laboratory study on the response of *T. castaneum* life stages exposed for different times to artificially created hypoxic conditions in hermetic containers.

## Materials and Methods

### Insects

*T. castaneum* were obtained from colonies maintained on a mixture of whole wheat flour and brewer’s yeast (95:5) used by the Stored Product Insect Rearing Facility at the Department of Entomology, Purdue University, West Lafayette, IN. Colonies were reared in a CARON Insect Growth Chamber (model 6025-1; Caron Products & Services, Inc., OH) at 27 ± 1°C, 60% relative humidity (RH), and at 16:8 (L:D) h photoperiod. The developmental life stages selected for the experiment were: egg (2 d), young larva (7 d), old larva (21 d), pupa (28 d), and freshly emerged unmated (selected and kept separately from the pupal stage), and mixed-sex adults (1–2 d after emergence).

### Experimental Preparation

Clear glass containers (30 ml) with plastic lids containing approximately 0.5 g of wheat flour added as food for the insects were used for each hypoxia treatment (Wheaton Glass Sample Bottle, CP Lab Safety, CA). Lids were perforated with several small holes to allow gas exchange when placed in chambers at different oxygen levels. Each container received a single *T. castaneum* (either an egg, young larvae, old larvae, pupae, or adult). For each combination of oxygen level (20.9, 8, 4, or 2% oxygen) and life stage treatments, 10 glass containers were prepared. We chose these low-oxygen levels (8, 4, and 2%) because they have been observed in hermetic bags during grain storage and have shown to preserve grain quality if the airtight conditions are maintained for several weeks.

### Hypoxia Treatment

Three chambers consisting of clear polycarbonate vacuum chambers 41.9 × 34.5 × 38 cm^3^, 35 liters capacity (Bel-Art - SP Science ware, NJ) were used to expose insects to different levels of hypoxia. The experiment was conducted at room temperature (23°C). Each chamber received 10 glass containers for each of the life stages of *T. castaneum* (single insect in each container), and the remaining 10 containers for each life stage used as control were kept at ambient oxygen levels (20.9%). Three of the polycarbonate chambers were randomly chosen for given levels of hypoxia (2, 4, or 8% oxygen levels) using a random sequence generator (Haahr 2002). The individual hypoxia levels in the chambers were created by flushing the air out of chambers while replacing it with nitrogen gas from a gas cylinder until the target concentration of oxygen in the chamber was attained. Oxygen content of the chambers was subsequently monitored every 3–4 h during the day and every 6 h during the night. An Oxysense 5250i oxygen reader device (Oxysense, Dallas, TX) was used in conjunction with fluorescent yellow Oxydots attached to the inner surfaces of the chamber. The Oxysense device is a noninvasive method for monitoring oxygen content of a sealed translucent container. Oxygen content was maintained within ± 0.25% of the target oxygen levels. If the oxygen levels elevated beyond ± 0.25%, nitrogen gas was pumped into the hypoxia chamber to restore the target level, and if the oxygen level declined below the target, the inlets in the chambers were opened for a short period to admit ambient air. Temperature and RH were monitored in the experimental room and inside the hypoxia treatment chambers using USB data loggers (Lascar, Erie, PA). Data on temperature and RH were recorded every 12 h.

### Exposure Duration and Experimental Run

The duration of exposure to each hypoxia condition was 1, 3, 5, 10, and 15 d. The hypoxia treatment for the different exposure days was conducted separately, i.e., when the individuals were taken out of the hypoxia chamber after exposing them for a given number of days, the treatment for another exposure day was initiated. The whole experiment was repeated three times (true replicates). This was done because each treatment combination (hypoxia levels and exposure periods) had 10 vials each containing a single individual of the life stage. The 10 vials for each treatment were held inside the same hypoxia chamber and were considered as pseudoreplicates.

### Data Collection

#### Adult Survivorship

At the end of each hypoxia treatment, the adults were removed from the hypoxia chamber and examined immediately. They were classified as live if they were capable of reflex movement and dead if they did not move when prodded with a probe. Data were recorded as a binary response. If an adult was alive, it was given the value 1, and a value of 0 if dead. Controls were classified in the same manner. To assess the effect of hypoxia on adults beyond the initial survivorship, all adults for each treatment group were placed into a single vial with food (*n* = 10). They were held in growth chambers (27 ± 1°C, 60% RH and 16:8 (L:D) h photoperiod) for 10 d. After 10 d, postexposure survivorship was determined using the same assessment as discussed earlier.

#### Immature Stage Experiment

Exposed eggs, young larvae, old larvae, and pupae were held for a maximum of 90 d posttreatment in the incubator (27°C, 60% RH) until any adult emergence had occurred. Adult emergence from exposed immatures of each treatment was recorded as a binary response, as earlier. Thus, if an adult emerged from an immature stage that had been exposed to hypoxia, it was recorded as 1. Similarly, if the immature stage failed to develop as an adult within 90 d, it was assigned a value of 0. In addition, the immature developmental time for *T. castaneum* was determined for the control and hypoxia-exposed young larvae. Only young larvae and no eggs were used for the developmental time studies because there was a high level of mortality in eggs exposed to hypoxia for even a short time. Young larvae exposed to 4 and 8% oxygen levels for 1 and 3 d had at least 80% hypoxia survival. Therefore, these two groups were chosen for further developmental time observations. The young larvae were then allowed to develop at ambient oxygen for 30 d posttreatment, after that they were checked every 3 d to record the time (d) of adult emergence.

### Statistical Analysis

Wald’s test was used to determine whether the covariance estimate of the random effect from three experimental runs was significantly different from 0 or not. If the experimental run showed no significant differences, data were pooled together for the variable experimental run for further analysis. To assess adult survivorship, logistic regressions were constructed using SAS PROC GLIMMIX Maximum Likelihood Estimation based on Laplace Approximation with the fixed effects of oxygen levels and exposure duration (SAS 2013). For the adult postexposure survivorship, the number of live adults in each treatment combination was determined and converted to percentage values and then transformed to angular values (Zar 2010). After that, the data were subjected to repeated measure ANOVA to determine the effects of oxygen levels, exposure duration, and postexposure days on adult survivorship. However, the data presented in the text are all untransformed mean percentage of live adults from three experimental runs.

Similarly, for the immature stages, logistic regression was constructed to model the proportion of adult emergence from immature stages using SAS PROC GLIMMIX Maximum Likelihood Estimation based on Laplace Approximation with the main effects of oxygen levels, exposure duration, and life stages. Data for the developmental period were analyzed using PROC GLM. Treatment means were separated using adjusted Tukey at *P* ≤ 0.05.

## Results

### Temperature and RH

There was no significant difference among temperatures and among RH for treatment kept inside and outside the hypoxia treatment chambers (data not shown). The mean and standard deviation of temperature for 20.9, 8, 4, and 2% oxygen levels were 24.01 ± 1.19, 23.69 ± 2.23, 23.56 ± 2.05, and 23.19 ± 1.66°C, respectively. Similarly, the mean and standard deviation of RH for 20.9, 8, 4, and 2% oxygen levels were 48.47 ± 8.61, 66.17 ± 15.69, 66.54 ± 12.54, and 61.96 ± 12.40, respectively.

### Adult Experiment

**Fig. 1.**
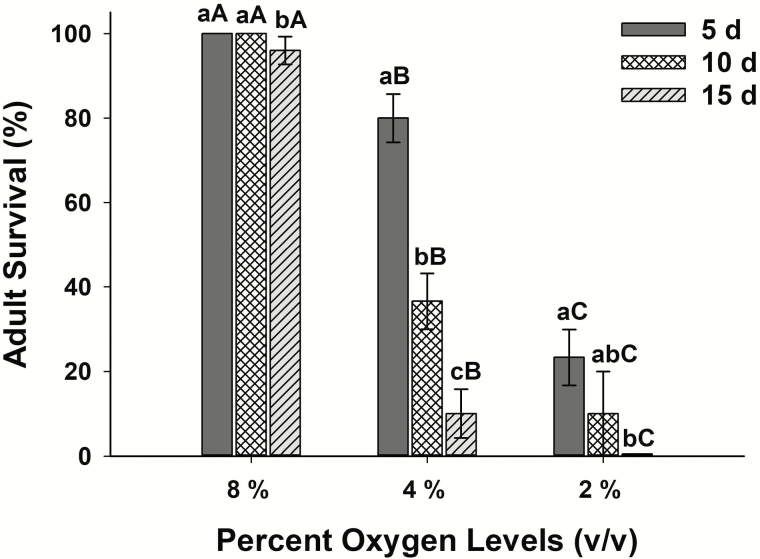
Percent *T. castaneum* adult survival after exposure to 2, 4, 8% hypoxia for 5, 10, and 15 d. The assessment was done immediately after returning the hypoxia-exposed adults to normoxia. Means for the same day among hypoxia levels (upper case letters) and within the same hypoxia level for different days (lower case letters) followed by the same letter are not significantly different (*P* ≥ 0.05, *n* = 30, adjusted Tukey). No mortality was recorded in insects kept at 20.9% (control), and those exposed to 2, 4, 8% oxygen levels for 1 and 3 d; hence the data were excluded from the GLIMX analysis.

The test of covariance parameters based on the likelihood ratio test showed that random effects from the experimental run were not significant (χ^2^ = 0.67, *P* = 0.21). Accordingly, the data were pooled together and analyzed for the effects of oxygen levels and exposure time. In the posttreatment assessment that begun immediately after returning insects to normoxia, we observed 100% adult survivals in control treatments and those exposed at all levels of hypoxia for 1 and 3 d. The data for 1 and 3 d and the controls were excluded from further analysis since there was no variation in the responses. We only used the hypoxia treatments of 2, 4, and 8% oxygen levels and for 5, 10, and 15 d to examine if there were posttreatment effects on adult survival. The GLIMMIX test of fixed effects showed that adult survival was significantly affected by 1) the oxygen levels (*F* = 18.51; df = 2, 259; *P* < 0.01), 2) exposure time (*F* = 3.39; df = 2, 259; *P* = 0.03), and 3) the interactions of oxygen levels × survival days (*F* = 32.29; df = 2, 259; *P* < 0.01). The probability of adult survival decreased as the oxygen levels decreased from 8 to 2% and likewise decreased as the length of exposure increased from 5 to 15 d. When exposed to 2% oxygen for 15 d, more than 99% of the exposed adults died. Exposure to 4% oxygen for more than 10 d resulted in more than half of the adults’ dead. In contrast, at 8% oxygen, over 90% of the insects survived even when exposed for 15 d (Fig. 1).

Post-exposure adult survival after 10 d was affected by the level of hypoxia (*F* = 446.50; df = 2, 58; *P* < 0.01), duration of exposure to hypoxia (*F* = 261.29; df = 4, 58; *P* < 0.01), and observation time after hypoxia treatment (d) (*F* = 43.70; df = 1, 58; *P* < 0.01) (Table 1). About 3.3–30% of the *T. castaneum* adults that initially survived the hypoxia treatment at 2% oxygen levels for 3–10 d were found dead 10 d later. *T. castaneum* adults that initially survived 4% oxygen for 1–15 d resulted in 3.3–13.3% death 10 d later. However, at 8% oxygen for 3 and 5 d, 3.3% mortality occurred 10 d later when compared with immediately after exposure (Table 1).

**Table 1.** Percent *T. castaneum* adult survival when exposed to 2, 4, and 8% oxygen levels for 1, 3, 5, 10, and 15 d (d) when assessed immediately after exposure and 10 d later (postexposure)

Percent adult survival (Mean ± SE)								
Exposure periods (days)	20.9% Oxygen		8% Oxygen		4% Oxygen		2% Oxygen	
	Immediately after exposure	10 d later	Immediately after exposure	10 d later	Immediately after exposure	10 d later	Immediately after exposure	10 d later
1	100Aa	100Aa	100Aa	100Aa	100Aa	96.7 ± 3.3Ab	100Aa	100Aa
3	100Aa	100Aa	100Aa	96.7 ± 3.3Ab	100Aa	90Bb	100Aa	70 ± 5.8Bb
5	100Aa	100Aa	100Aa	96.7 ± 3.3Ab	80 ± 5.7Ba	66.7 ± 3.3Cb	23.3 ± 6.7Ba	13.3 ± 6.7Cb
10	100Aa	100Aa	100Aa	100Aa	36.7 ± 6.6Ca	26.7 ± 3.3Db	10 ± 10Ba	6.7 ± 3.3Cb
15	100Aa	100Aa	96.7 ± 3.3Aa	96.7 ± 3.3Aa	10 ± 5.7Da	6.6 ± 3.3Ea	0Ca	0Da

Data presented is the mean ± SE of percent survival of 10 adults for each treatment replicated three times. Means within the same oxygen level, within column among exposure days (upper case letter) and between columns for same exposure day (lower case letter) followed by the same letter are not significantly different (*P* ≥ 0.05, *n* = 30, adjusted Tukey).

### Immature Stages

The test of covariance parameters based on the likelihood ratio test showed that random effects from the experimental run were not significant (χ^2^ = 0.53, *P* = 0.23). The test of fixed effects revealed that adult emergence from exposed immatures was significantly affected by 1) the insect developmental life stage exposed to hypoxia (*F* = 769.12; df = 3, 2318; *P* < 0.01), 2) oxygen levels (*F* = 810.17; df = 3, 2318; *P* < 0.01), 3) exposure time (*F* = 348.22; df = 4, 2318; *P* < 0.01), and 4) the interactions of life stages × oxygen levels × exposure time (*F* = 142.69; df = 34, 2318; *P* < 0.01). Eggs were the most susceptible immature life stage to hypoxia treatment (Table 2). Oxygen levels of 4% or less for 3 d or more killed all eggs. A hypoxia level of 4% oxygen or less for at least 10 d killed over 75% of the young larvae, older larvae, and pupae. About 80% of the young larvae, old larvae, and pupae exposed to the 8% oxygen levels survived regardless of exposure duration (Table 2).

**Table 2.** Percent live adult emergence from the immature life stages of *T. castaneum* exposed to 2, 4, 8 and 20.9% levels of oxygen for 1, 3, 5, 10, and 15 d

Oxygen levels	Exposure periods (days)	Percent live adult emergence (Mean ± SE)			
		Egg	Young larvae	Old larvae	Pupae
20.9 %	1	100 ± 0 Aa	100 ± 0Aa	100 ± 0Aa	100 ± 0Aa
	3	100 ± 0Aa	100 ± 0Aa	100 ± 0Aa	100 ± 0Aa
	5	95.5 ± 4.2Aa	100 ± 0Aa	100 ± 0Aa	100 ± 0Aa
	10	100 ± 0Aa	100 ± 0Aa	100 ± 0Aa	100 ± 0Aa
	15	96.7 ± 3.3Aa	100 ± 0Aa	100 ± 0Aa	96.7 ± 3.3Aa
8%	1	73.4 ± 8.2Ac	90.1 ± 5.5Ab	96.7 ± 3.3Aab	100 ± 0Aa
	3	36.6 ± 8.9BCb	96.7 ± 3.2Aa	100 ± 0Aa	100 ± 0Aa
	5	53.3 ± 9.3Bc	96.6 ± 3.3Aa	83.4 ± 6.8Bb	100 ± 0Aa
	10	23.3 ± 7.8Cb	96.7 ± 3.2Aa	90.1 ± 5.5ABa	93.4 ± 4.6Ba
	15	6.6 ± 4.5Db	73.4 ± 8.2Ba	83.4 ± 6Ba	80.1 ± 7.4Ca
4%	1	56.7 ± 9.2Ab	80.1 ± 7.4Aa	93.4 ± 4.6Aa	86.7 ± 6.2Ba
	3	4 ± 1.1Bc	90.1 ± 5.5Aa	73.4 ± 8.2Bb	100 ± 0Aa
	5	3.3 ± 3.3BCd	29.9 ± 8.5Bc	56.7 ± 9.3Ba	70.1 ± 8.5Ca
	10	3.3 ± 3.2BCb	23.3 ± 7.9Ba	9.9 ± 5.5Ca	19.9 ± 7.4Da
	15	0 ± 0Cb	6.6 ± 4.8Ca	0 ± 0Db	6.7 ± 4.6Ea
2%	1	50 ± 9.3Ab	39.9 ± 9.1Ab	90 ± 5.5Aa	93.4 ± 4.6Aa
	3	0 ± 0Bc	0 ± 0Bc	16.6 ± 6.8Bb	66.7 ± 8.7Ba
	5	0 ± 0Bb	3.3 ± 3.3Bab	6.7 ± 4.6Ba	3.3 ± 3.2Cab
	10	0 ± 0Ba	0 ± 0Ba	0 ± 0Ca	0 ± 0Ca
	15	0 ± 0Ba	0 ± 0Ba	0 ± 0Ca	0 ± 0Ca

Eggs (2 d), young larvae (7 d), old larvae (21 d), and pupae (28 d) were exposed to 2, 4, 8, 20.9% oxygen levels for 1, 3, 5, 10, and 15 d. After hypoxia treatments, the immature stages were held for a maximum of 90 d at normoxia to observe adult emergence. Means within the same oxygen level, among exposure days (upper case letters) and among immature life stages (lower case letters) followed by the same letter are not significantly different (*P* ≥ 0.05, *n* = 30, adjusted Tukey).

**Fig. 2.**
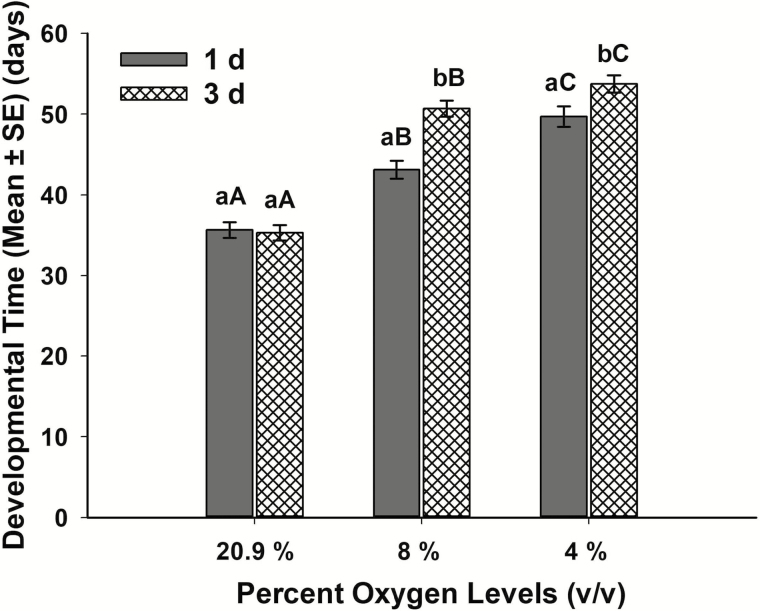
Total time required by young larvae of *T. castaneum* (7 d old) to reach adulthood when exposed to 4%, 8%, and atmospheric levels of oxygen (20.9%) for 1 and 3 d. Means for the same day among hypoxia levels (upper case letters) and within the same hypoxia level for different days (lower case letters) followed by the same letter are not significantly different (*P* ≥ 0.05, *n* = 30, adjusted Tukey).

Furthermore, the two-way ANOVA to determine the effects of oxygen levels and exposure time on the development duration of young larvae to adult emergence showed that developmental time was significantly affected by the oxygen levels, exposure days, and their interactions. The young larvae-to-adulthood developmental time increased by 14 d at 4% and by 8 d at 8% oxygen level compared with control when young larvae were exposed to hypoxia for 1 d. Similarly, when exposed to hypoxia for 3 d, the developmental time increased by 18 d at 4% oxygen and by 15 d at 8% compared with the control (Fig. 2).

## Discussion

### Hypoxia Exposure of Adults

Exposing *T. castaneum* adults to 2% oxygen for 10 d killed 90% of them. Increasing the exposure period to 15 d resulted in near total mortality (>99%). However, to reach 90% mortality at 4% oxygen, adults required exposure for 15 d. On the other hand, exposure to 8% oxygen for 15 d produced <5% mortality. Our findings are consistent with those of previous researchers who reported that most stored product insects are sensitive to the oxygen levels <5%, (Donahaye 1990; Donahaye et al. 1992, 1996; Viebrantz et al. 2016; Yan et al. 2016; Njoroge et al. 2017). Low RH inside hermetic containers (<30%) are known to cause desiccation in the stored product insects resulting in rapid mortality (Navarro 1978). RH inside the hermetic containers remained above 60% in all of the hypoxia chambers (compared with 48% RH at room condition), hence, did not contribute to the insect mortality in the present study.

Depletion of oxygen levels in hermetic containers is a gradual, biologically driven process. Depending on the initial insect infestation level, it can take several days or even weeks to reach <5% oxygen levels. It would enhance the value of hermetic storage if there were a mechanism to accelerate oxygen depletion in the hermetic container. On the other hand, in many cases, oxygen levels within hermetic storage systems are depleted rapidly reaching <5% within a few days. This happens when the infestation level is relatively high and when the ambient temperature is warmer. These conditions promote a more rapid rate of insect metabolism, and in some cases, respiration of the grain (Williams et al. 2016, Kharel et al. 2018). In addition, limited oxygen supply may affect insect behavior besides survivorship. Hypoxia has been shown to reduce oviposition, progeny development, body mass, and longevity of the insects (Spratt 1979, Cheng et al. 2012, Yan et al. 2016), all of which slow overall population growth.

Further evidence of the sublethal effects of hypoxia is our present observations of additional mortality of *T. castaneum* adults at 10 d posttreatment. This suggests that many of the adults that responded positively to the test probing (i.e., survive) immediately after hypoxia treatments of low oxygen levels such as 2 or 4% were, in fact, moribund and died by 10 d posttreatment. The survival of *T. castaneum* adults 10 d after posttreatment appeared to decrease with increased duration of exposure to 2% oxygen, though there was no change to insects exposed for 1 and 15 d. Insect exposed to 2% oxygen for 1 d were all alive while those exposed for 15 d were all dead. Exposure to 2% for 3 d resulted in 30% posttreatment death 10 d later. Though all insects were alive immediately after 2% exposure for 3 d, *T. castaneum* adults were severely affected resulting in additional death 10 d later. Further investigations of insect behavior and physiology of the surviving adults would shed valuable light on the still largely unknown sublethal effects of hypoxia on insects.

### Hypoxia Effects on Immature Stages

Our study shows that the different immature life stages of *T. castaneum* respond differently to hypoxia. The egg was the most susceptible stage, with 100% mortality to 2% oxygen when exposed for at least 3 d. Yan et al. (2016) found that exposing eggs of *C. maculatus* to 2% oxygen for 2 d suppressed adult emergence by over 80%, and increasing the exposure time to 3 d, reduced adult emergence by >98% compared with eggs exposed to normal ambient oxygen levels. Oxygen levels of 4% or less for 3 d or more killed all eggs. These findings corroborate results by Tunc and Navaro (1983) that showed complete mortality when eggs were exposed to 2 and 4% oxygen levels for 96 h (4 d) at three RH (20, 50, and 95%). Further, this study concluded that the various RH affected egg mortality slightly. In our current study, we believe that recorded RH of 48–66% were optimal enough and had no effects on the mortality of eggs. For other juvenile stages, exposure to hypoxia of 2% oxygen for at least 15 d was critical to arrest adult emergence of exposed individuals. According to Woods et al. (2005), adult and juvenile insects possess efficient systems to regulate oxygen flux and water balance, but eggs lack sensory systems to monitor hypoxia, thereby making the egg stage the most sensitive to hypoxic environments. Interestingly, many studies in the past have shown *T. castaneum* eggs to be the most tolerant stages to conventional pest control methods such as grain fumigants used in crop storage protection systems (Bell et al. 1998, Walse et al. 2009). Hence, storing grains and processed food commodities in hermetic bags could contribute to control population development of *T. castaneum*.

The pupal stage was the least sensitive of the immature stages to hypoxia. This may be due to depressed respiratory metabolism during pupation (Navarro 2006). Further studies are needed to understand the exposure time required to achieve complete mortality of all developmental stages of *T. castaneum* above 2% oxygen level. Nevertheless, when exposed to 2 or 4% oxygen levels for 15 d, over 93% of the individuals of all developmental stages succumbed. As with the adult stage, immature stages were less susceptible to 8% oxygen compared with lower levels, as over 75% of individuals of all developmental stages survived 15 d of exposure to this level of hypoxia. Even so, a short exposure (<3 d) to 8% oxygen levels caused marked developmental delays in immature *T. castaneum*. Previously Loudon (1989) reported that the larvae of *Tenebrio molitor* L. (Coleoptera: Tenebrionidae) exposed to 10% oxygen levels molted 12.3 times compared to 5.8 times at normoxia, thereby supporting the assumptions of hypoxia-related developmental delay in insects. In this study, we found that exposure to 8% oxygen for 1 and 3 d increase development time by 21 and 43.5%, respectively, when compared exposure with normoxia. Exposure to 4% oxygen for 1 and 3 d increase the development time by 39.4 and 52.1%, respectively, when compared exposure with normoxia. This delay will have the effect of retarding population growth, thereby helping make the hermetic system more effective in controlling the numbers of insect present.

In conclusion, *T. castaneum* eggs, the most tolerant stage to the conventional fumigants, can be controlled using hermetic storage with oxygen level of 2% for at least 3 d; while other juvenile stages would require exposure to this same oxygen level for 10 d. Adults of *T. castaneum* require exposure period of 15 d at 2% oxygen level to produce complete mortality. However, hermetic containers with oxygen maintained at 4% would require 15 d to kill eggs and old larvae but not the other insect life stages. Oxygen levels of 8% may not directly contribute to the death of adults and immature stages of *T. castaneum* but can increase the developmental time of the immature stages, thereby slowing the rate of population expansion. Maintaining oxygen levels inside hermetic containers at 2% for at least 15 d will help control *T. castaneum*.

## References

[CIT0001] Bailey S. W 1965 Airtight storage of grain: its effect on insect pests, IV. *Rhyzopertha dominica* (E) and some other Coleoptera that infest grain. J. Stored Prod. Res. 1: 25–33.

[CIT0002] Baoua I. B., Amadou L., Ousmane B., Baributsa D., and Murdock L. L.. 2014 PICS bags for post-harvest storage of maize grain in West Africa. J. Stored Prod. Res. 58: 20–28.

[CIT0003] Baributsa D., Lowenberg-DeBoer J., Murdock L. L., and Moussa B.. 2010 Profitable chemical-free cowpea storage technology for smallholder farmers in Africa: opportunities and challenges. Julius-Kühn-Archiv. 425: 1046.

[CIT0004] Bell C. H., Savvidou N., and Wontner-Smith T. J.. 1998 The toxicity of sulfuryl fluoride (Vikane®) to eggs of insect pests of flour mills, pp. 345–350. *In* Zuxun J., Quan L., Yongsheng L., Xianchang T., and G. Lianhua (eds.), Proceedings, 7th International Working Conference on Stored Product Protection, 14–19 October 1998, Beijing, P.R. China.

[CIT0005] Campbell J. F., and Runnion C.. 2003 Patch exploitation by female red flour beetles, *Tribolium castaneum*. J. Insect Sci. 3: 1–8. 15841236 10.1093/jis/3.1.20 PMC524659

[CIT0006] Cheng W. N., Lei J. X., Ahn J. E., Liu T. X., and Zhu-Salzman K.. 2012 Effects of decreased O_2_ and elevated CO_2_ on survival, development, and gene expression in cowpea bruchids. J. Insect Physiol. 58: 792–800. 22387498 10.1016/j.jinsphys.2012.02.005

[CIT0007] Donahaye E 1990 Laboratory selection of resistance by the red flour beetle, *Tribolium castaneum* (Herbst), to an atmosphere of low oxygen concentration. Phytoparasitica. 18: 189–202.

[CIT0008] Donahaye E., Zalach D. and Rindner M.. 1992 Comparison of the sensitivity of the development stages of three strains of the red flour beetle (Coleoptera: Tenebrionidae) to modified atmospheres. J. Econ. Entomol. 85: 1450–1452.

[CIT0009] Donahaye E., Navarro S., Rindner M. and Azrieli A.. 1996 The combined influence of temperature and modified atmospheres on *Tribolium castaneum* (Herbst) (Coleoptera: Tenebrionidae). J. Stored Prod. Res. 32: 225–232.

[CIT0010] Epidi T. E., and Odili E. O.. 2009 Biocidal activity of selected plant powders against *Tribolium castaneum* Herbst in stored groundnut (*Arachis hypogaea* L.). Afr. J. Environ. Sci. Technol. 3: 1–5.

[CIT0011] Haahr M 2002 Random sequence generator http://www.random.org/ sform.html.

[CIT0012] Jagadeesan R., Collins P. J., Daglish G. J., Ebert P. R., and Schlipalius D. I.. 2012 Phosphine resistance in the rust red flour beetle, *Tribolium castaneum* (Coleoptera: Tenebrionidae): inheritance, gene interactions and fitness costs. Plos One. 7: e31582. 22363681 10.1371/journal.pone.0031582 PMC3283673

[CIT0013] Kharel K., Mason L. J., Williams S. B., Murdock L. L., Baoua I. B., and Baributsa D.. 2018 A time-saving method for sealing Purdue Improved Crop Storage (PICS) bags. J. Stored Prod. Res. 77: 106–111. 29899581 10.1016/j.jspr.2018.04.002 PMC5992327

[CIT0014] Lee B. H., Lee S. E., Annis P. C., Pratt S. J., Park B. S., and Tumaalii F.. 2002 Fumigant toxicity of essential oils and monoterpenes against the red flour beetle, *Tribolium castaneum* Herbst. J. Asia-Pac. Entomol. 5: 237–240.

[CIT0015] Loudon C 1989 Tracheal hypertrophy in mealworms: design and plasticity in oxygen supply systems. J. Exp. Biol. 147: 217–235.

[CIT0016] Martin D. T., Baributsa D., Huesing J. E., Williams S. B., and Murdock L. L.. 2015 PICS bags protect wheat grain, *Triticum aestivum* (L.), against rice weevil, *Sitophilus oryzae* (L.) (Coleoptera: Curculionidae). J. Stored Prod. Res. 63: 22–30.

[CIT0017] Murdock L. L., Margam V., Baoua I. B., Balfe S., and Shade R. E.. 2012 Death by desiccation: effects of hermetic storage on cowpea bruchids. J. Stored Prod. Res. 49: 166–170.

[CIT0018] Navarro S 1978 The effects of low oxygen tensions on three stored-product insect pests. Phytoparasitica. 6: 51–58.

[CIT0019] Navarro S 2006 Modified atmospheres for the control of stored-product insects and mites, pp. 105–146. *In* J. W. Heaps (ed.), Insect management for food storage and processing, AACC International, St. Paul, MN.

[CIT0020] Navarro S., Donahaye E., and Fishman S.. 1994 The future of hermetic storage of dry grains in tropical and subtropical climates, pp. 130–138. *In* Highley E., Wright E. J., Banks H. J., and B. R. Champ (eds.), Proceedings, 6th International Working Conference on Stored-Product Protection, 17–23 April 1994, Canberra, Australia, CAB International, Wallingford, United Kingdom.

[CIT0021] Njoroge A. W., Mankin R. W., Smith B. W., and Baributsa D.. 2017 Effects of hermetic storage on adult *Sitophilus oryzae* L. (Coleoptera: Curculionidae) acoustic activity patterns and mortality. J. Econ. Entomol. 110: 2707–2715. 29045682 10.1093/jee/tox260 PMC5946933

[CIT0022] SAS Institute 2013 SAS 9.2 for Windows. SAS Institute, Cary, NC.

[CIT0023] Spratt E. C 1979 The effects of a mixture of oxygen, carbon dioxide and nitrogen in the ratio 1: 1: 8 on the longevity and the rate of increase of populations of *Sitophilus zeamais* Mots. J. Stored Prod. Res. 15: 81–85.

[CIT0024] Tunc I., and Navarro S.. 1983 Sensitivity of *Tribolium castaneum* eggs to modified atmospheres. Entomol. Exp. Appl. 34: 221–226.

[CIT0025] Viebrantz P. C., Radunz L. L., and Dionello R. G.. 2016 Mortality of insects and quality of maize grains in hermetic and non-hermetic storage. Rev. Bras. Eng. Agríc. Ambient. 20: 487–492.

[CIT0026] Walse S. S., Tebbets S., and Leesch J. G.. 2009 Ovicidal efficacy of sulfuryl fluoride to stored-product pests of dried fruit, pp. 60:1–2. *In* Proceedings, Methyl Bromide Alternatives and Emissions Research Conference, 29 October–10 November, San Diego, CA.

[CIT0027] White G. G 1988 Field estimates of population growth rates of *Tribolium castaneum* (Herbst) and *Rhyzopertha dominica* (F.) (Coleoptera: Tenebrionidae and Bostrychidae) in bulk wheat. J. Stored Prod. Res. 24: 13–22.

[CIT0028] Williams S. B., Murdock L. L., Kharel K., and Baributsa D.. 2016 Grain size and grain depth restrict oxygen movement in leaky hermetic containers and contribute to protective effect. J. Stored Prod. Res. 69: 65–71. 27990031 10.1016/j.jspr.2016.06.006 PMC5146321

[CIT0029] Woods H. A., Bonnecaze R. T., and Zrubek B.. 2005 Oxygen and water flux across eggshells of *Manduca sexta*. J. Exp. Biol. 208: 1297–1308. 15781890 10.1242/jeb.01525

[CIT0030] Yan Y., Williams S. B., Baributsa D., and Murdock L. L.. 2016 Hypoxia treatment of *Callosobruchus maculatus* females and its effects on reproductive output and development of progeny following exposure. Insects. 7: 26. 10.3390/insects7020026 PMC4931438 27322332

[CIT0031] Zar J. H 2010 Biostatistical analysis, p. 944, 5th ed. Pearson Prentice-Hall, Upper Saddle River, NJ. ISBN: 0321656865.

[CIT0032] Zettler L. J., and Cuperus G. W.. 1990 Pesticide resistance in *Tribolium castaneum* (Coleoptera: Tenebrionidae) and *Rhyzopertha dominica* (Coleoptera: Bostrichidae) in wheat. J. Econ. Entomol. 83: 1677–1681.

